# Experiences of Older Adults, Physiotherapists, and Aged Care Staff in the TOP UP Telephysiotherapy Program: Interview Study of the TOP UP Interventions

**DOI:** 10.2196/53010

**Published:** 2024-02-07

**Authors:** Rik Dawson, Heidi Gilchrist, Marina Pinheiro, Karn Nelson, Nina Bowes, Cathie Sherrington, Abby Haynes

**Affiliations:** 1 Institute for Musculoskeletal Health, Sydney Musculoskeletal Health Sydney Local Health District The University of Sydney Camperdown Australia; 2 Whiddon Sydney Australia; 3 Uniting AgeWell Melbourne Australia

**Keywords:** physiotherapy, telehealth, telephysiotherapy, exercise, aged care, qualitative methods, behavior change, technology, virtual care

## Abstract

**Background:**

Telehealth provides opportunities for older adults to access health care. However, limited research exists on the use of telehealth within aged care services, particularly regarding physiotherapy-led fall prevention and mobility programs. Understanding the experiences and interactions of older adults, physiotherapists, and aged care service providers is crucial for the scale-up and sustainability of such essential programs. The TOP UP study, a hybrid type 1 effectiveness-implementation randomized controlled trial in aged care, used a supported multidisciplinary telephysiotherapy model to motivate older adults to engage in exercises to improve mobility and reduce falls.

**Objective:**

This qualitative substudy aims to achieve 2 primary objectives: to describe the experiences and acceptability of the TOP UP intervention for older people, physiotherapists, and aged care support workers and managers and to gain an in-depth understanding of program implementation.

**Methods:**

A purposive recruitment strategy was used to select 18 older adults who participated in the TOP UP intervention, ensuring variation in age, gender, residential status (home or residential aged care), geographic location, and cognitive levels. In addition, 7 physiotherapists, 8 aged care support workers, and 6 managers from 7 different aged care provider partners participated in this study. Semistructured interviews were conducted to explore stakeholders’ experiences with the TOP UP program, gather suggestions for improvement, and obtain insights for the future implementation of similar telephysiotherapy programs. The interview framework and coding processes were informed by behavior changes and implementation frameworks. Data were analyzed using an abductive approach, informed by 2 behavioral change theories (Capability, Opportunity, Motivation, and Behavior Model and Self-Determination Theory) and the Nonadoption, Abandonment and Challenges to the Scale-Up, Spread and Sustainability of Health and Care Technologies framework.

**Results:**

All participants (n=39) reported high levels of acceptability for the TOP UP program and cited multiple perceived benefits. The thematic analysis generated 6 main themes: telephysiotherapy expands opportunity; tailored physiotherapy care with local support enhances motivation; engaging, older adult–friendly educational resources build capability; flexible reablement approach fosters autonomy; telephysiotherapy is safe, effective, and acceptable for many; and organizational commitment is required to embed telehealth. The motivation to exercise was enhanced by Zoom’s convenience, use of tailored web-based exercise resources, and companionable local support.

**Conclusions:**

This study highlights the inherent value of telephysiotherapy in aged care, emphasizing the need for investment in staff training, local support, and older adult–friendly resources in future telephysiotherapy iterations. TOP UP represents a convenient and flexible web-based care model that empowers many older adults to receive sustainable, high-quality care precisely when and where they need it.

**Trial Registration:**

Australian New Zealand Clinical Trials Registry (ANZCTR) ACTRN 1261000734864; https://anzctr.org.au/Trial/Registration/TrialReview.aspx?ACTRN=12621000734864

## Introduction

### Background

The proportion of older people in the population is increasing worldwide. From 2020 to 2050, the number of older people aged ≥60 years will double to 2.1 billion, representing 22% of the world’s population [1]. By 2050, the number of people aged ≥80 years is expected to triple to 426 million [1]. Older people experience poor mobility and higher rates of falls than younger people, leading to significant morbidity, mortality, and poor quality of life [2-4]. Poor mobility and falls are 2 of the biggest cost drivers in hospital and aged care services [2,5]. In 2021, a total of 10.7% of people aged ≥65 years living in the Organization for Economic Co-operation and Development countries received long-term care, either at home or in care facilities, costing these governments 1.5% of the gross domestic product [6,7]. Aged care spending has increased over the last 15 years in most Organization for Economic Co-operation and Development countries, and population aging will continue to increase the demand on stretched health care systems [7].

Strong evidence supports the effectiveness and cost-effectiveness of physiotherapy-led exercise programs for enhancing mobility and reducing falls in aged care settings [8]. However, the Australian Royal Commission into Aged Care highlighted significant barriers to accessing mobility-promoting and fall prevention interventions delivered by allied health professionals, such as physiotherapists [9]. Qualitative evidence suggests that (referred and defined in this manuscript as telephysiotherapy) could be a feasible, acceptable, and effective approach for delivering mobility and fall prevention programs to older adults living in the community [10]. Telephysiotherapy could be particularly advantageous in increasing access and convenience for people with travel constraints and mobility limitations or who live in regional and remote areas [11].

Telerehabilitation that has provided telephysiotherapy has been found to have similar effectiveness compared with in-person rehabilitation services for community-dwelling older people, and it shows no increased risk of adverse events [12]. However, there is no evidence supporting the effectiveness, cost-effectiveness, and implementation feasibility of telephysiotherapy for improving mobility, reducing falls, and enhancing the quality of life for older adults receiving aged care services in their homes or residential aged care.

Telehealth is currently being used in aged care, but there is limited guidance on how best to implement it [13]. Hybrid effectiveness and implementation research has been shown to accelerate research translation into clinical practice [14]. Implementation research explores the experience of a complex intervention such as telehealth and its relationship to other factors, such as intervention engagement and adherence, perceived effectiveness, acceptability, and self-efficacy, which can support implementation translation [15].

### The TOP UP Trial

TOP UP is designed to provide a scalable solution for delivering physiotherapy exercise interventions via telehealth to improve mobility, reduce falls, and enhance the quality of life in aged care. The TOP UP program was developed in collaboration with our aged care partners, physiotherapists, and aged care service users and their caregivers. A series of workshops identified potential facilitators to improve older people’s engagement with technology and motivation to exercise. The program’s co-design was also influenced by behavior change models, such as Self-Determination Theory (SDT) [16] and the capability, opportunity, motivation, and behavior (COM-B) framework [17].

TOP UP is investigating synchronous and asynchronous care to optimize both personalized health care and self-directed exercise [18] in aged care settings. It involves the delivery of real-time physiotherapy assessments through videoconferencing (synchronous telehealth) using the Zoom app (Zoom Video Communications Inc) by older adults receiving aged care services at home or in residential care. These service users are given access to evidence-based exercise videos on the TOP UP website and the StandingTall app (asynchronous telehealth) to support their exercise program. Each participant has the weekly support of a trained aged care worker to help them access the Zoom app and follow the exercise program. The outcomes being measured include effectiveness (mobility, falls, and quality of life), cost-effectiveness, and implementation measures (acceptability, reach, fidelity, dose delivered, and adoption).

The program is being tested in a hybrid type 1 effectiveness-implementation randomized controlled trial. Older adults were screened by their aged care service providers. Eligibility criteria included the age of older people (≥65 years); possessing sufficient physical, sensory, cognitive, and English language skills to participate; and having individual consent or consent from the person responsible. Those with terminal or unstable illness, with severe dementia, having participated in a similar physiotherapy program in the previous year, or being unable to walk 10 m were excluded from the study. A total of 242 participants were recruited from a screening pool of 1348 aged care service users (older people).

A total of 242 participants (120 per group) will provide 80% power to detect a 0.9 point between-group difference in 12-point Short Physical Performance Battery test scores at 6 months (assuming SD 2.8, *P*=.05, and 20% dropouts) [19]. A 0.5-point between-group difference in the Short Physical Performance Battery test was considered clinically significant. This sample size was expected to be sufficient to detect between-group differences of 10% to 15% for the secondary outcome measures. Quantitative data analysis is expected to be completed in 2024.

Participants randomized to the intervention group received 10 videoconference physiotherapy sessions over 6 months using the Zoom app and received an individualized balance and strength exercise program. These exercise programs are based on the World Health Organization 2020 guidelines on physical activity and sedentary behavior [20] and the Otago exercise programs [21]. Existing aged care support staff, called “coaches,” have been trained to supervise participants to access the technology and provide “hands-on” exercise support once per week with the assistance of exercise videos designed by the research team. The waitlist control group receives a 3-month version of the program once the intervention period at each site is completed.

To inform the successful development of programs such as TOP UP, it is essential to examine not just if but how and why TOP UP worked (or not) and what strategies could best improve it. The aim of this paper was to use interview data to provide detailed insights into the experiences of older people, physiotherapists, coaches, and aged care managers with the telephysiotherapy intervention. The objective was to understand how contextual factors mediate the delivery of the TOP UP program and to produce transferable lessons for the potential use of future telephysiotherapy in aged care [22].

## Methods

### Study Design and Context

This study used a qualitative, descriptive approach through semistructured one-on-one interviews [23]. Qualitative description is increasingly used in conjunction with effectiveness and implementation trials and aims to present a straightforward description of participants’ experiences [24]. The analysis is grounded in the participants’ own words, making the results accessible to vulnerable groups, valid, highly translatable, and useful for refining interventions [25]. Qualitative description sits within a constructivist paradigm, considers multiple meanings, and recognizes that the research process is never neutral [26]. To strengthen the research rigor, we included triangulated data sources (by drawing on perspectives of different stakeholder groups) and a reflective discussion of emergent findings among the multidisciplinary research team [27].

### Conceptual Framework

**Figure 1:**
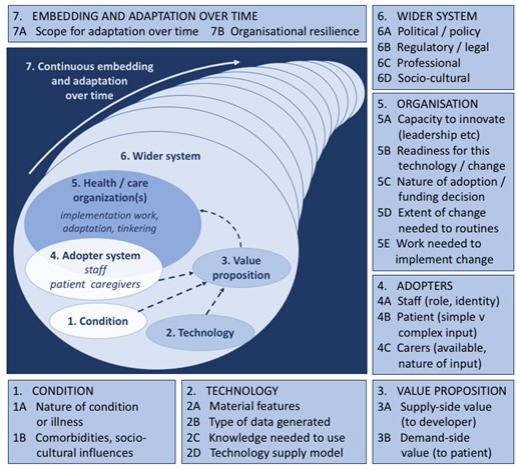
The Nonadoption, Abandonment and Challenges to the Scale-Up, Spread and Sustainability of Health and Care Technologies framework.

We used 2 behavior change theories, COM-B and SDT, and the Nonadoption, Abandonment and Challenges to the Scale-Up, Spread and Sustainability of Health and Care Technologies (NASSS) framework to provide a conceptual “lens” to inform data collection and analysis [28]. The COM-B model of behavior change proposes that to engage in a behavior such as exercise (B), a person must be physically and psychologically capable (C) and have the opportunity (O) to engage in the behavior, as well as the motivation to do so (M). COM-B simplifies complex factors and recognizes that to modify behavior, we need to address at least one of these components [17]. SDT focuses on the motivation underpinning behavior change, positing that effective programs must support autonomy, competency, and relatedness [16]. The NASSS framework is an evidence-based, theory-informed, and pragmatic framework that can help predict and evaluate the success of a technology-supported health program. It consolidates multiple implementation frameworks, targeting key issues relating to the implementation and uptake of telehealth at the microlevel of individual staff and consumers, the mesolevel challenges of organizational engagement and adoption, and macrolevel policy and regulatory factors (Figure 1) [28].

### Recruitment and Data Collection

At the initial TOP UP recruitment, all aged care service users, coaches, physiotherapists, and aged care managers received an information sheet inviting them to participate in an interview for this qualitative study. After participants read the informational letter and confirmed their interest in participating in an interview, they received an informed consent letter to be signed by themselves or their person responsible before the interview appointment. A list of potential aged care service users and their coaches and physiotherapists was created in consultation with 3 of our aged care partners (Ashfield Baptist Homes, Whiddon, and Uniting AgeWell).

A purposive recruitment strategy was used to select 18 older adults who participated in the TOP UP intervention, ensuring variation in age, gender, residential status (home or residential aged care), geographic location, and cognitive levels. All 39 participants contacted agreed verbally and in writing before and on the day of the Zoom interview. None of the participants declined to participate in the interviews. The interviews were conducted 3 to 6 months after the interviewees commenced the program. Recruitment was stopped at the point when data adequacy had been reached, that is, when we judged that we had sufficient rich data across our purposive sample with which to answer our research questions [29].

The interview guide was created in consultation with the wider research team and representatives from our aged care partners (Multimedia Appendix 1). Interview questions targeted concepts from the COM-B model, SDT, and NASSS framework (described earlier). Specific questions explored the relative value of the different components of TOP UP (eg, Zoom, exercise videos, and the level of support provided). Questions regarding its implementation and effectiveness were also included. We also asked interviewees to identify local and potential system-wide barriers and facilitators to the successful adoption of telephysiotherapy, such as TOP UP and other programs aimed at delivering fall prevention and mobility programs in aged care. We used open-ended questions and active listening to confirm our understanding of the interviewees’ perspectives. RD and KN conducted the interviews individually on Zoom. They were involved in the delivery of the program, so to reduce sociability bias, interviewees were encouraged to critique the TOP UP program and its implementation to identify improvements [30].

Aged care participants had a family member or someone familiar to them from their aged care organization that was not their coach to assist them with connection to the Zoom app and to support them through the interview. The participants were reminded that they could stop the interview at any time. No repeat interviews were conducted. RD and KN completed memos after the interviews and met to discuss the data and the emerging thematic content. Interview audio recordings were automatically transcribed using Zoom’s free transcription service, and transcripts were corrected by RD. Transcripts were not returned to the participants.

### Data Analysis

The transcripts and interview field notes were uploaded to NVivo 12 (Lumivero) for data management and coding [31]. The transcripts were coded by one researcher (RD) using an abductive analytical approach. RD drafted an initial thematic framework drawing on emergent themes in the data and was informed by domains from the NASSS framework. In total, 2 researchers (RD and AH) tested and refined the coding framework on 2 manuscripts, adding codes and modifying existing codes from inductively identified concepts in the data. RD coded the remaining data. RD, CS, and AH met regularly to discuss emergent codes and themes.

Recurrent themes were generated from reading across the coded data and reviewed against concepts from SDT [16] and the COM-B framework [17,29] to understand how aspects of the TOP UP program influenced exercise engagement. An early overview of the findings was discussed with all coauthors and our consumer representative to explore a wider range of possible thematic interpretations and to help ensure that we had answered our research questions, including considering the implications of our findings. Disagreements were resolved through discussion. The criteria for reporting qualitative research was used as a reporting checklist (Multimedia Appendix 2) [32].

### Ethical Considerations

Ethics approval for this qualitative substudy was included in the TOP UP study approval granted by the Ethics Review Committee at the Sydney Local Health District Research Ethics and Governance Office, Concord, Australia (approval number CH62/6/2021-009). The trial was registered with the Australian New Zealand Clinical Trials Registry (ACTRN 1261000734864).

## Results

### Participants

In total, 39 people participated in semistructured interviews: 18 (46%) aged care service users who completed the TOP UP program, 7 (18%) aged care physiotherapists, 8 (21%) coaches, and 6 (15%) aged care managers. Interviews took an average of 19 (range 8-53) minutes. These service users were aged from 70 to 93 (median 87.5) years at the start of the intervention; 11 (61%) were female and 7 (39%) were male; 11 (61%) used a 4-wheeled walking frame to walk and 7 (39%) did not need a walking aid to walk; 7 (39%) lived in metropolitan cities in New South Wales and 11 (61%) lived in rural or remote areas in New South Wales and Victoria; 6 (33%) had mild to moderate cognitive impairment and 12 (77%) had no cognitive impairment; all had multiple comorbidities (median 7, range 2-11); and 10 (56%; median 1, range 1-7) had one or more falls in the last 12 months. The median Technology Readiness Index score was 2 out of 5 (range 1-3.9), which classified aged care users as technology *avoiders,* people who tend to have a high degree of resistance and a low degree of motivation to use technology [33]. A total of 4 (22%) aged care service users had used phone-based telehealth before with their general practitioner, but none had used a videoconferencing app such as Zoom before the study or used telehealth to receive physiotherapy.

Of the 7 physiotherapists interviewed, 4 were based in metropolitan areas and 3 were based in rural areas. A total of 4 coaches supported aged care service users from residential aged care, and 4 coaches supported aged care service users from home aged care. In addition, 1 aged care manager worked at a remote residential aged care site, 1 managed a rural residential aged care site, and 4 were home care managers from rural areas.

### Main Findings

#### Overview

Our qualitative analysis revealed that all interviewees found the TOP UP program to be acceptable and would recommend similar telephysiotherapy programs to other older people receiving aged care services. Thematic analysis generated 6 key themes related to the experiences of TOP UP. We also compiled evidence of these experiences to identify and manage emergent possibilities, uncertainties, and interdependence that could guide the adoption of telephysiotherapy in aged care using the NASSS framework. Quotations were used to illustrate each theme. We annotated the quotes for anonymity with aged care service users referred to as P1, P2, and so on, and other stakeholders are descriptively described.

#### Theme 1: Telephysiotherapy Expands Opportunity

Theme 1 highlights the expanded opportunities for accessing the physiotherapy that TOP UP provided. TOP UP minimized barriers to physiotherapy access related to travel and associated costs:

Travel in country areas is just too hard and having telehealth in the home makes it so easy to do. I can’t do a 70 km round trip – it is too expensive.

P10

This was also echoed by service users whose significant disabilities created access barriers:

Because of my health, there’s no way I can go out to see a physio. One, I’ve got to get someone to take me, like a relation, or pay someone to take you, it’s not practical. It’s hard to park anywhere near the physio, you’ve got to walk, so by the time you get to the physio you’re exhausted.

P1

All the physiotherapists and aged care service managers indicated that telehealth could deliver physiotherapy care efficiently, improving opportunities for older adults receiving aged care services to receive physiotherapy where and when they need it:

Some people need to be able to see a physio quickly, and we can provide telehealth services quickly, it is so efficient.

Home care physiotherapist, metro

TOP UP had the greatest impact on rural and remote services, especially in areas where telehealth has the potential to address chronic health inequity issues related to workforce shortages:

Our town has a physio that visits once a month. Recently, one of our residents had a fall so I called the clinic and found out that we can’t get an appointment to take our resident to see a physio for 6 weeks, if someone has a fall like this, we just can’t wait six weeks. Telehealth really helps us.

Residential manager, remote

#### Theme 2: Tailored Physiotherapy With Local Support Enhances Motivation

Regular local support was identified by all interviewees as important for enhancing older people’s confidence to try the exercise program and to support their motivation to “stick with” the program, including coping with TOP UP’s increasing challenge over time. Many interviewees across the 4 stakeholder groups explained that it was not just physical and technological support that the coaches offered (eg, providing stand-by assistance while performing balance exercises and managing the Zoom app) but also companionship and emotional support:

I can’t get out much and I began to look forward to the weekly session with my care worker as I really appreciated the support, she gave me to do something positive for my health. My coach understood what was going on in my life and she gave me the confidence to keep doing the exercises.

P12

All the physiotherapists interviewed indicated that the coaches’ “hands-on” support was vital to the success of the program, as it helped to build capability and confidence. Importantly, TOP UP was a tailored program in which physiotherapists were able to modify exercises according to the individual needs of each service user, mirroring the person-centered approach typical in-person physiotherapy sessions. This tailored approach was particularly important to aged care service users whose health changes required program adaption:

My physio understood that I needed a break when I had some surgery, but was ready for me when I got back home and quickly helped me regain the fitness that I had lost in the hospital.

P18

The TOP UP program used technology and behavior change techniques to maximize program adherence. Zoom provided physiotherapists with a platform to deliver individualized real-time health coaching and goal setting, which has been shown in the literature to increase participant adherence [34]. Interview data indicated that these techniques were being used across program implementation, consistent with the behavior change theory:

Physiotherapists start with building external motivation by setting goals, by encouraging them, and highlighting their progress we help them develop internal motivation to keep going. If we can motivate them internally, half the job is done, and exercise will become a routine and a lifestyle habit.

Home care physiotherapist, metro

Further evidence emerged that these techniques were having the desired effect:

Their motivation seemed to improve when they reached their goals, and they wanted to keep on trying. Their motivation is the most important thing.

Residential coach, metro

However, it was the combination of live tailored physiotherapy with enthusiastic and companionable local support that seemed to both develop confidence and underpin motivation:

The individual sessions on Zoom were important, so I could ask some questions about how I was doing, and having my physio give me some individual feedback was important for my confidence to keep exercising. My coach has made it possible, and her support has been great, she is so lively, and she exercises with me which makes it fun, we had such a laugh, she keeps me motivated, and she takes the monotony out of it. If you are not having fun, it is not worth it.

P10

The previous quote also highlights the vital role of enjoyment in exercise and how this can be enhanced with a trained support worker acting as a coach. This may be especially important for engagement in the TOP UP program, given that many of the physiotherapists interviewed suggested that telehealth requires more time to develop a therapeutic alliance. Therapeutic alliance refers to how people experience the empathy of clinicians, and research shows that a strong therapeutic alliance is connected to positive treatment adherence and results in physical therapy [35]:

It’s not until you get to the fourth or fifth telehealth session that people start getting to really know you and feel like you can be an advocate for them. I think that telehealth does allow for a personal connection which adds to exercise adherence.

Physiotherapist, home care, rural

TOP UP physiotherapists and coaches’ person-centered approach to goal setting, highlighting progress during the program and celebrating any achievements, seemed to enhance the aged care service user’s motivation to exercise:

TOP UP helped me care about my future before I just didn’t care. I loved the way the physio explained things to me carefully, so I understood. I really appreciate having the support worker exercise with me and reinforce what to do, and how to do the exercises. All of this made me feel like I mattered and now I can walk further, do my shopping, which is a big improvement.

P12

#### Theme 3: Engaging, Older Adult–Friendly Educational Resources Build Capability

Interviewees expressed enthusiasm for the instructional videos that were designed to support high-quality independent exercises throughout the program. The videos incorporated exercises modeled by an older person, slow-paced dialog in a warm conversational style, natural lighting to maximize visibility, minimal visual distractions, and gentle humor, all of which seemed to increase exercise engagement by older people. In the following quotes, 2 participants describe the importance of “seeing” another older person in web-based videos:

It was great to see an older person do the exercises, really motivating to see someone my age doing the program. The videos were at the right pace, and I like how they got harder over time. It was fun.

P17

The exercise videos are motivating because I feel like I am doing it with someone – it’s interactive and fun. Following a book can be boring.

P5

Many stakeholders commented positively on the video design that incorporated slow demonstration and simple dialog that aimed to teach aged care service users how to perform safe and effective home exercise:

The physiotherapist in the videos demonstrated the exercises slowly and explained things easily. I was really surprised how the residents were able to follow everything without any help.

Residential coach, metro

Having online exercise resources really helps because people aren’t familiar with exercise techniques, they can follow their prescribed video and it helps keep their exercise dose up.

Home care physiotherapist, regional

Many of the physiotherapists interviewed commented that the TOP UP program was complex and challenged participants to navigate different apps and printed resources such as exercise diaries while using Zoom. They suggested that simpler telephysiotherapy programs (or simpler mechanisms for accessing program components) could be developed to enhance the user experience and minimize program dropout. One physiotherapist commented:

I think having an easy to navigate, no fuss system where our clients can look up an exercise, record their exercise program and any problems they may have had, a fall, etc. I think an app where physiotherapists could get access to this information easily during a session and to help prepare for another session would be useful.

Home care physiotherapist, rural

#### Theme 4: A Flexible Reablement Approach Fosters Autonomy

TOP UP is designed to encourage older adults to take a lead in their program planning; flexibility is emphasized, including choices about what resources to use (printed and web-based) and what skills they wish to develop that would enable them to engage in activities they found most important:

I liked how it started easily, and I moved my way up the program. There is structure to the program, and you commit to it. I often plan to do a session but if something comes up, I make an appointment with myself to make sure I do it another time.

P16

I liked that I could stop and start the videos according to my own needs on the day.

P1

This can be described as a reablement approach, and the physiotherapist and coaches were encouraged to build the aged care service users’ physical capability and support them in transferring their new skills to access other activities in their community independently:

Residents lack enough physical activity here, sometimes we are short staffed, and sometimes the staff don’t have time to help. It was great to see our residents on the TOP UP program improve their mobility and begin to walk to different activities on their own.

Residential manager, rural

All stakeholders valued the reablement approach, and it was reported that TOP UP seemed to be a catalyst for reablement, as many of their clients began to engage in more socialization with friends and families and embrace other physical activities as they became stronger and more mobile:

Physios and coaches can work together to ensure that the participant becomes independent and autonomous in their use of telehealth and do more exercise as the program progresses. As they improved, we had discussions with them and their coach about how they could do more outdoor walking.

Home care physiotherapist, rural

I was surprised about the other quality of life benefits of telehealth, talking to their physio on zoom, seeing their support workers in this new way, learning how to get out and about in the community, all seemed to reduce social isolation, which is so important for our customers.

Home care manager, rural

#### Theme 5: Telephysiotherapy Can Be Safe, Effective, and Acceptable for Many

Most interviewees regarded TOP UP as a safe, effective, and acceptable program. Interviewees reported positive physical and quality of life improvements:

I think it’s fabulous. I wouldn’t have imagined that I would be given the opportunity to get physio. Physically, I can walk further. My breathing is better. I’m stronger, it gives you more independence.

P1

Telehealth has not only helped my customer’s strength, mobility and coordination, but it seemed to help their overall quality of life, they seemed happier and more confident to walk.

Residential care manager, rural

Many interviewees reported that the combination of physiotherapist-led instructional exercise videos and supervision by trained support workers increased the safety of the TOP UP:

I think having a physio run exercises in the videos gives the intervention more authority, frees up my time to motivate the residents and keep them safe.

Residential coach, remote

I think having the care worker there with the client to help set up Zoom, hold the iPad, and angling the video so I can see them clearly makes the program safer and more successful.

Home care physiotherapist, rural

However, TOP UP was not considered to be suitable for all aged care service users. All stakeholders agreed that telehealth presents challenges for frail clients in residential aged care, who often have higher levels of mobility and sensory and cognitive disability. A total of 2 cognitive and sensory impaired aged care service users found using Zoom to “see” their physiotherapist frustrating and as a result, pulled out of the program:

First of all, not all dementia residents get used to it, and second, people with hearing and vision problems struggle to follow.

Residential aged care manager, rural

Some physiotherapists would hesitate to use telehealth without local support for those aged care service users with high fall risks:

For people who are mostly independent I wasn’t worried, but if I did have someone who was who was frailer and there was no one there with them I was worried they might fall.

Home care physiotherapist, regional

Some aged care service users and managers suggested that although telephysiotherapy is a good secondary option, they would still prefer in-person physiotherapy, especially for older adults with more complex needs:

I prefer a blend of face-to-face physio and telehealth. I need some hands-on physio from time to time to manage the arthritis in my back, but I liked the telehealth program because I could follow the physio exercise videos at home, it was so convenient.

P18

It appears that a hybrid model that incorporates a blend of face-to-face physiotherapy and web-based exercise resources, such as exercise videos, was viewed as particularly acceptable for those with significant health challenges:

I don’t know if someone with severe dementia or disabilities would be able to access telehealth. I also think a lot of clients would like a hybrid telehealth model starting with a face-to-face assessment.

Home care physiotherapist, rural

Finally, our screening process uncovered many technological hesitations and potential telehealth data concerns that prevented the recruitment of many potential aged care service users into the TOP UP trial:

There is some hesitancy around technology use due to recent cybersecurity anxiety in the community- for example the Optus and Medibank breaches.

Home care manager, rural

#### Theme 6: Organizational Commitment Is Required to Embed Telephysiotherapy

Interviewees explained that considerable organizational commitment is required to embed telehealth programs such as TOP UP in aged care. Sufficient investment is required to train staff, conduct more meetings with their physiotherapy service providers to plan for the development of a new service, such as telephysiotherapy, prioritize TOP UP sessions within busy service schedules, and, where necessary, direct funds toward supportive technology. Some coaches and physiotherapists commented that the use of devices such as large iPads and smart televisions enhanced telehealth engagement by improving the visibility and hearing experience of service users:

Zoom worked well when we connected the iPad to the TV, we were able to turn the volume of the TV up so the resident could hear better. It also gives a bigger picture as well, so they can see the physio better.

Residential care coach, rural

However, such equipment can be costly, and telehealth-specific funding was raised by physiotherapy, aged care managers, and coaches as a key condition for ongoing sustainability of telephysiotherapy in aged care:

I think that maybe there needs to be funding support. Telehealth is an important and easy way to increase access and uptake. One physio could service several homes in a full-time caseload.

Residential care physiotherapist, rural

TOP UP required 3 people to be available for appointments (the older person, their coach, and the physiotherapist on Zoom); thus, scheduling was more challenging than 2-person face-to-face health care interactions:

There are always challenges whenever it comes to scheduling, especially during COVID when we were short of staff. But if you have a good relationship with your physiotherapy provider, who is responsive to time slot suggestions, then our scheduling team could work their magic and get it all booked.

Home care manager, rural

Training was provided to older adults to increase their confidence using an iPad, our website, and relevant apps (Zoom and StandingTall). Coaches were trained to increase their level of comfort by navigating the TOP UP website and Zoom. Physiotherapists were trained to deliver effective telephysiotherapy assessments using Zoom and provided strategies to enhance relationship development with older adults and their coaches. All interviewees highlighted this training as an important factor in overcoming “telehealth hesitancy” both for service users and program providers:

There was a lot of telehealth hesitancy at the beginning, but with education they slowly got quite comfortable in doing it.

Home care physiotherapist, metro

There is a need to have some general training so we [physiotherapists] know how to use it [telehealth technology] effectively: make sure your voice is coming through, how to pace instruction so our clients understand us. The coaches and customers need training to know how to set up a shot, to make sure that they are visible to ensure that the client becomes independent and autonomous in their use of telehealth.

Home care physiotherapist, rural

The aged care service managers also noted the challenge of training adequate numbers of care workers to facilitate TOP UP and ensure that the coaches are safe and competent:

There is a need to train a large proportion of our support workforce so that we have more trained staff who know how the program works, how to use technology and how to supervise our customers safely.

Home care manager, rural

Some aged care service managers and physiotherapists indicated that more frequent and more detailed web-based exercise training programs would be useful to improve the skill level of a wider group of support staff:

It is very important to have lots of staff trained. For example, if the regular coach is sick, another staff member could take over and keep the program going.

Home care manager, rural

All stakeholders indicated the need for specific investment into better internet connectivity to ensure the sustainability of future telephysiotherapy programs:

I’ve found is there are still a lot of places in rural Australia where older people don’t have fast Internet, they don’t have smart TVs, or they don’t have the technology that metro places have. People are ready to engage with telehealth, but there’s no infrastructure in rural areas.

Residential physiotherapist, metro

### Implementation Guidance Through the Lens of the NASSS Framework

TOP UP appears to be well positioned for sustainable adoption, and learnings from this study have informed the translation of telephysiotherapy services by our aged care partners into practice. Table 1 uses the NASSS framework to help explain TOP UP’s successes and failures and explore the facilitators required to embed similar telephysiotherapy programs in aged care.

**Table 1:** An overview of TOP UP implementation guidance in relation to the Nonadoption, Abandonment and Challenges to the Scale-Up, Spread and Sustainability of Health and Care Technologies framework domains.

Domain	Definition of domain	Implementation guidance derived from study findings	Illustrative quotes from interviewees
The condition	The suitability of the participant’s attributes/needs and their interaction with the intervention.	TOP UP is suitable for aged care service users with mobility challenges who can walk short distances. It is not suitable for those with significant sensory and cognitive disability	“Someone with severe dementia or severe disabilities would not be able to have that skill to access telehealth*.*” [Residential coach, rural]
The technology	Technical features related to the usability of telehealth and its support requirements.	TOP UP requires access to the internet via an iPad or similar device. Aged care service users do not need technological skills due to the weekly support they received from trained care workers to help them use the iPad, navigate Zoom, and access exercise videos on a website. However, basic technological skills were often developed, which increased autonomy.	“One of my clients is really good with technology but other clients need my help to turn on the iPad and follow the program.” [Residential coach, metro]
The value proposition	The value proposition of telehealth for upstream end users (aged care service providers) and downstream users (physiotherapists and their clients).	All stakeholders saw telephysiotherapy as a valuable addition because of its convenience and perceived effectiveness, especially for those with poor mobility or who are living in rural or remote areas. The value proposition for telehealth to treat musculoskeletal pain is less as stakeholders prefer a more “hands-on” experience. A hybrid model would add value for some.	“Telehealth would save us time and travel and help us to see more people.” [Home care physiotherapist, regional]
The adopter system	The ongoing investment required to support the telehealth intervention and the ongoing acceptability of stakeholders.	TOP UP requires consistent investment in training, human (physio, coaches), and physical infrastructure (devices, fast internet, senior-friendly exercise resources) to create sustainable success. However, high levels of system support are likely to be reinforced as positive returns on investment due to their perceived positive impacts on mobility and well-being.	“TOP UP is more than just a fall prevention program, it offers a truly reablement focus where our clients can build their strength and balance and get out into the community again. I think many of our clients could benefit from telehealth.” [Home care manager, rural]
The organization	An organization’s capacity to embrace the telehealth intervention and the supports required to establish and maintain it as a viable service offering.	Not all aged care services chose to participate in TOP UP because of the perceived burden of working with technology. Providers who joined TOP UP wanted to investigate telehealth’s impact on access to fall prevention and mobility programs, in areas where there are physiotherapy shortages. Providers offered considerable support via technology provision, extra administration support for scheduling of telephysiotherapy sessions, and enough care workers to support the program.	“I was surprised at how easy telehealth was to get started. We gave the clients an iPad and the assistance the care workers gave them was important to help them engage with telehealth. Our scheduling team are fantastic, and they managed to solve the scheduling challenges really well.” [Home care manager, rural]
The wider context	The wider organizational and policy impacts on telehealth uptake and sustainability.	Stakeholders agreed that funders need to provide telehealth-specific funding and education for interventions such as TOP UP to reduce technology hesitation and improve telehealth systems that enhance its adoption and sustainability.	“I feel that people would be greatly advantaged if there was a separate pocket of funding for allied health so that we could afford to deliver ongoing telehealth” [Home care manager, rural]

## Discussion

### Principal Findings

This study, which included participants such as older adults, physiotherapists, aged care support workers, and managers in the TOP UP trial, offers valuable insights. Our thematic analysis identified key factors for the telephysiotherapy program’s acceptability, including advice from physiotherapists, consistent support from trained care workers, older adult–friendly web-based exercise resources, and a flexible reablement approach. The interview data supported multiple themes, suggesting that the synergistic integration of these ingredients within the TOP UP contributed to its high acceptability. The discussion explores the impact of single components and emphasizes their combined contribution to TOP UP’s acceptability.

### TOP UP Study Is Acceptable

Acceptability is an important consideration in the design and implementation of complex health care interventions, such as TOP UP [36]. Our findings align with the increasing body of literature indicating acceptance of telehealth among older adults in community settings despite high levels of technology hesitation [37,38]. A cohort study of a telehealth program incorporating physiotherapy for rural older adults found that telehealth was safe (no adverse events) and feasible (average telehealth attendance 85%) [38]. A 2021 cross-sectional survey of health care providers further affirmed increasing telehealth acceptability over time among homebound older adults [39].

### Barriers and Facilitators Related to Telehealth Adoption

TOP UP identified several barriers and facilitators that enabled aged care service users to overcome high levels of technology hesitation and, if appropriately addressed, could improve the translation of telehealth programs into aged care [40]. TOP UP’s qualitative findings are reflected in the literature, which demonstrate that barriers related to this population’s innate technology hesitation and greater sensory, physical, and cognitive impairments could be addressed by the provision of local support, internet-connected devices, fast internet, and appropriate telehealth training can mitigate these barriers [39,41,42].

A recent US survey of physician providers of homebound older adults during the COVID-19 pandemic revealed that a significant proportion of their patients were technology avoidant [39] (only a third of their patients had used video-based telehealth before, 310/873, 35.5%). Among patients who had not used telehealth before, providers deemed that one in 4 (153/563, 27.5%) of their patients would not be able “interact over video” due to cognitive or sensory impairments. This survey found other barriers: providers lacked knowledge of their patients’ internet connectivity, and participants faced financial constraints in obtaining internet plans and were unable to pay for internet plans or video-capable devices. Similar findings emerged in the TOP UP, where most trial participants had limited access (10/18, 56%) to video-capable devices, limited telehealth experience (4/18, 22%), and low telehealth readiness (Technology Readiness Index 2 out of 5). Addressing barriers related to the purchase of telehealth infrastructure and providing local support can facilitate wider acceptance within aged care settings.

A recent qualitative exploration of factors influencing acceptability in dementia management revealed that videoconferencing had potential benefits over in-person appointments by improving access to care for those with mobility limitations and reducing the stress associated with clinic appointments [43]. A crucial insight from this study emphasized the necessity of technical support and telehealth training involving information on how to access and use different telehealth apps and tips for setting up the video camera for maximum visibility. Similarly, another study examining telehealth’s role in enhancing oncology care for older adults emphasized that appropriate technology training integrated into the screening process and program delivery could enhance telehealth adoption [44]. These studies align with TOP UP’s findings that emphasized the delivery of appropriate education at screening and recruitment to reduce technology avoidant behaviors, preprogram technology training to support adoption, and training to troubleshoot any emerging technology issues to enhance sustainability.

TOP UP demonstrated that behavior change training for physiotherapists and coaches in health coaching techniques, motivational interviewing, and collaborative goal setting can facilitate telehealth adoption. Behavior change training has been shown to increase therapeutic alliance and enhance exercise program outcomes in other studies [45]. A strong therapeutic alliance has been identified as a crucial facilitator in previous telehealth interventions [46]. In our study, physiotherapists, coaches, and aged care service users found telehealth suitable for effective behavioral change coaching and suggested that specific training on skills to enhance therapeutic alliance is important to augment telehealth acceptability. Specific examples included targeted training on using Zoom emojis to acknowledge client achievements and building a personal connection through virtual tours of the older person’s home and garden. However, they noted that establishing a successful therapeutic alliance through telehealth demands more time compared with in-person sessions, potentially increasing program costs.

### Telehealth Can Provide Key Ingredients for Behavior Change

TOP UP was co-designed to incorporate the COM-B model to create positive behavior changes related to exercise adherence [17]. Recent data from the Australian Institute for Health and Welfare have shown the critical significance of addressing insufficient physical activity in older individuals, given their 50% contribution to 2.5% of the overall disease burden in Australia [47]. Consequently, increasing motivation and opportunities for exercise in this demographic is crucial in mitigating the adverse health consequences stemming from sedentary behavior [19] and in supporting the efficiency of the health care system [48]. TOP UP’s tailored approach and use of older adult–friendly resources appeared to increase the capability (C) of older adults to exercise. The program provided increased opportunities (O) for exercise by facilitating increased access to physiotherapists. Furthermore, TOP UP heightened motivation (M) through its reablement approach, goal-setting mechanisms, and cultivation of enjoyment via companionable coaching [16].

The TOP UP program strategically incorporated the principles of SDT to promote increased exercise adherence. According to SDT, intrinsic motivation thrives when individuals perceive a sense of autonomy and control over their activities [49]. Our study findings suggest that the aged care service users valued the opportunity to regain independence through self-directed exercise. The TOP UP program effectively nurtured feelings of competence through its personalized and progressive exercise routines program, fostering a sense of relatedness through local support and the rapport established during the telephysiotherapy sessions that actively promoted enjoyment. This observation aligns with the systematic review by Teixeira et al [50] on SDT and exercise adherence, affirming the positive correlation between intrinsic motivation, enjoyment, personal achievement, and heightened program acceptability.

Our study has provided insights into the potential explanatory effects of the social learning theory by Bandura [51] and Motivational Theory of Role Modeling in supporting the high acceptability of TOP UP. The social learning theory by Bandura [51] underscores the significance of observation and imitation in driving behavior change. When individuals perceive the modeled behavior as valuable, and the model possesses an admired status while being relatable, the likelihood of behavioral change increases. In this context, physiotherapists, esteemed as exercise professionals in the community [52], played a crucial role in enhancing the perceived value of the TOP UP program. Furthermore, the Motivational Theory of Role Modeling highlights another critical aspect of TOP UP’s acceptability [53]. Many interviewees emphasized the importance of including older adults as role models in exercise videos. Both theories suggest that the inclusion of older role models was a pivotal factor inspiring behavioral change, explaining the positive reception of TOP UP exercise videos.

### Scale-Up and Sustainability of Telephysiotherapy in Aged Care

Telehealth has emerged as a prominent method for implementing scalable health care interventions, a trend that has intensified during the COVID-19 pandemic [54]. However, the challenge of sustaining these programs is pressing, as is evident from reports of high participant attrition rates in telehealth-led exercise programs [55]. Successfully delivering cost-effective exercise programs to frail older adults with multiple comorbidities in the aged care environment is challenging and complex, demanding significant resourcing [8]. Insights gained from the NASSS framework [28] underscore the need for careful screening of older adults for telephysiotherapy participation and the provision of targeted training to all stakeholders to enhance its feasibility. Our analysis indicates that while TOP UP was acceptable, a hybrid model of virtual care that combines in-person initial assessments, subsequent synchronous telephysiotherapy sessions for program progression, and the integration of local support and older adult–friendly web-based exercise resources may further increase telephysiotherapy uptake and sustainability in aged care.

Although the cost-effectiveness analysis of TOP UP is pending, our qualitative observations indicate that establishing telephysiotherapy programs requires substantial investment in both physical and human infrastructure. The telehealth literature discusses the critical role governments play in developing policies and guidelines to foster telehealth adoption [56]. Our interviews revealed a consensus on the need for dedicated funding for telehealth to enhance adoption and sustainability.

### Strengths, Limitations, and Future Studies

This qualitative study had several strengths. It triangulates empirical data relating to the uptake and sustainability of telephysiotherapy in aged care from 4 perspectives: older adults receiving physiotherapy within aged care services, physiotherapists, trained support workers who deliver the intervention, and aged care managers who are charged with case management and overseeing aged care service resource allocation and delivery. Our partnerships with aged care providers and their ongoing input in the research have enabled us to develop a deep understanding of how the TOP UP program was delivered in aged care and, if proven effective, this will speed up its translation into wider practice [14].

Qualitative research serves as a valuable tool for refining program design, deepening insights into the outcomes of quantitative research, and offering valuable guidance for enhancing the implementation of complex interventions such as telephysiotherapy in aged care [26]. In this study, we adopted a broad sampling strategy aimed at delivering a rich description of diverse intervention experiences, enlisting the perspectives of 18 older adults encompassing a range of sociodemographic characteristics distributed across 10 distinct sites. Moreover, the inclusion of independent physiotherapists, separate from both the aged care service partners and the research team, in our study design may have reduced potential social desirability bias, enhancing the credibility of our findings [30].

Several limitations of this study necessitate careful consideration. TOP UP excluded participants from culturally and linguistically diverse backgrounds and thus presented a notable gap in our understanding of their experiences. To address this gap, future trials that prioritize the inclusion of culturally and linguistically diverse communities are required. In addition, although the interviewee cohort was purposefully selected to encompass maximum variation, it is essential to acknowledge that this pool primarily consisted of individuals who voluntarily participated in the trial, potentially predisposing them to higher levels of exercise engagement and receptiveness to telehealth. Consequently, this may limit the generalizability of our findings.

Several aged care service users and coaches were interviewed by either a physiotherapist or aged care service provider who delivered the program. This can lead to social desirability biases, which may undermine the credibility of the study results [57]. Given this context, aged care service users and staff might hesitate to openly share negative experiences with their interviewers despite the research team’s assurances that their feedback would have no bearing on their ongoing care or employment status. To mitigate this bias, interviewers made concerted efforts to positioning themselves as eager learners, actively encouraging interviewees to share their “insider” perspectives on quality improvement and expressing genuine appreciation for any criticism offered. Future larger-scale mixed methods studies should be designed to enhance research quality and further explore the impact of telephysiotherapy uptake and sustainability in aged care while carefully addressing social desirability bias.

This study suggests a need for the development of simplified telephysiotherapy exercise programs to facilitate greater adoption in aged care. A recent scoping review conducted in 2021, examining the barriers and facilitators to the use of telehealth by older adults, found several impediments associated with current technology, including challenges related to small screens, text size, small icons, insufficient color contrast between text and background, and complex functionality [58]. The review also identified ease of use as a key facilitator of telehealth adoption. Some TOP UP stakeholders interviewed indicated a preference for simplified functionality tailored to this demographic. Respondents expressed a desire for telehealth programs that incorporated TOP UP program features such as Zoom, exercise diaries, and videos, into one user-friendly application. These findings advocate for further research aimed at enhancing the user experience.

### Conclusions

This interview study explored the program experiences of aged care service users, physiotherapists, and aged care staff involved in the TOP UP trial, a telehealth-led exercise program designed to improve mobility, reduce falls, and enhance quality of life. All stakeholders indicated high program acceptability, underscored by its safety, and perceived effectiveness. The thematic analysis uncovered key insights: TOP UP’s provision of convenient access to physiotherapy services for aged care recipients, the positive impact of tailored physiotherapy coupled with local support on exercise motivation, the effectiveness of engaging older adult–friendly resources in fostering program adherence, and the facilitation of greater independence through a flexible reablement approach. This study emphasizes the importance of sustained organizational commitment for the successful implementation of telephysiotherapy programs, such as TOP UP, highlighting the need for training and external funding to ensure telephysiotherapy’s adoption and sustainability.

## Appendix

Multimedia Appendix 1 Semistructured interview guide.

Multimedia Appendix 2 The Consolidated Criteria for Reporting Qualitative Research (COREQ) checklist.

## Abbreviations

COM-B: capability, opportunity, motivation, and behavior
NASSS: Nonadoption, Abandonment and Challenges to the Scale-Up, Spread and Sustainability of Health and Care Technologies
SDT: Self-Determination Theory

## Data Availability

The data sets generated during this study are not publicly available because of the confidentiality promised to the participants as part of the informed consent process. However, data sets are available from the corresponding author upon reasonable request.
