# Supplementary material for: Experiences of Older Adults, Physiotherapists, and Aged Care Staff in the TOP UP Telephysiotherapy Program: Interview Study of the TOP UP Interventions
Source: JMIR Aging. 2024 Feb 7;7:e53010. doi: 10.2196/53010 (PMC10882472; doi:10.2196/53010)
Supplement: Multimedia Appendix 1 [file aging_v7i1e53010_app1.docx]

**Appendix 1: Semi structured interview guide**

Questions were omitted or modified as appropriate for the type of participant or the participant’s background.

**Part A. Reasons for participation**

1. Have you ever used telehealth before? What were your views about telehealth before you started the program?
2. What made you decide to take part in the TOP UP study?

**Part B. Experiences of participation**

1. What was your *experience of the TOP UP program overall?*
2. What are the *best aspects* of the TOP UP physiotherapy telehealth program? Specifically:
3. What did you think about *using the iPad*? How much assistance was required to use it?
4. Can you tell me what it was like to receive /give *advice from the physiotherapist*?
5. Did you use the *exercise booklet*? Was it useful?
6. Did you enjoy *the one-to-one exercise session with your TOP UP coach*? How are important is this help?
7. Did you follow the *video programs* from our website? What did you think about them?
8. Did you use *the StandingTall app*? What did you like or dislike about it?
9. What didn’t work so well for you in the TOP UP program? What would you change about it?
10. We want to understand how a program like this fits in with people’s lives (or, perhaps, doesn’t fit in). Is there anything that gets in the way or makes it difficult for you take part?
11. Exercise is important, but it can also feel like hard work. What helped you/your client stick with the exercises and why do you think it helped? Was there any part of the program that that made it easier to keep going or was particularly motivating?
12. How has COVID has affected how we do things? Did it affect your ability to do your exercise?

**Part C. Impacts**

1. Do you/your client think anything has changed physically or mentally because of taking part in the physiotherapy program so far? Have you noticed any changes in your/their balance, how far you/they can walk, or your/their independence?
2. What are your views about physiotherapy telehealth now? Do you think it has benefits for you/your client?

**Part D. What works?**

1. We have some ideas about why people would choose to see their physiotherapist using telehealth. Can I share these ideas with you and get your views on them. We think:
2. That using telehealth increase people’s *access to physiotherapy.* Has that been your experience?
3. The *tailoring of the physiotherapy program* is important. So, our physiotherapists try to create exercise according to everyone’s abilities, taking account of your health needs, using different combinations of exercise handouts and online exercise classes to follow. Do you think we tailored the program to you and was this important?
4. Have you become *independent* doing your exercise program? If yes what helped you? If no, why not?

**PART E. Maintenance**

1. Thinking about the future now
2. What do you feel will happen with your/their physiotherapy program over the next 12 months? Do you see yourself keeping up with your exercises?
3. Would you/your client like to keep doing the online exercise programs? If not, why? What would help you with that? For example: would you like to own your own iPad?
4. Would you like to see the Coaches and physio regularly? How often? How much would you be prepared to pay for your physio telehealth?

**Final thoughts** Is there anything else you can tell us that might help us to improve telehealth physiotherapy programs like this and to support older people to be as active as possible and improve their health?

***Thank you so much for helping us with our research. It’s been really helpful to hear about your experience.***
