# Supplementary material for: Experiences of Older Adults, Physiotherapists, and Aged Care Staff in the TOP UP Telephysiotherapy Program: Interview Study of the TOP UP Interventions
Source: JMIR Aging. 2024 Feb 7;7:e53010. doi: 10.2196/53010 (PMC10882472; doi:10.2196/53010)
Supplement: Multimedia Appendix 2 [file aging_v7i1e53010_app2.docx]

| **Appendix 2: Consolidated criteria for reporting qualitative research (COREQ) checklist** | | |
| --- | --- | --- |
|  | | |
| **No** | **Item** | **Description** |
| **Domain 1: research team and reflexivity** | | |
| Personal Characteristics | | |
| 1 | Interviewer | RD and KN conducted the interviews |
| 2 | Credentials | RD PhD candidate, KN extensive research experience |
| 3 | Occupation | RD Physiotherapist, KN Aged Care Executive Manager |
| 4 | Gender | RD male, KN female |
| 5 | Experiences | RD completed post graduate qualitative course, KN 10 year+ conducting clinical research into aged care |
| Relationship with participants | | |
| 6 | Relationship | RD conducted all the baselines assessment, KN is an executive manager with participants aged care service provider |
| 7 | Participant knowledge of the interviewer | Participants were aware of the research aims |
| 8 | Interviewer characteristics | All participants were made aware the RD was doing his PhD thesis investigating telehealth physiotherapy in aged care, all relevant participants were aware the KN was investigating the implementation of telehealth physiotherapy for possible business integration |
| **Domain 2: Study Design** | | |
| Theoretical framework | | |
| 9 | Method | Qualitative description |
| Participant selection | | |
| 10 | Sampling | Purposive |
| 11 | Approach | Phone |
| 12 | Sample size | 39 |
| 13 | Non-participation | Nil |
| Setting | | |
| 14 | Data collection | Participants home |
| 15 | Presence of non-participants | Trained aged care support worker |
| 16 | Description of sample | Aged care service users – home and residential aged care |
| Data Collection | | |
| 17 | Interview guide | Interview guide attached and piloted in previous study |
| 18 | Repeat Interview | No |
| 19 | Audio recording | Zoom audio transcription was used |
| 20 | Field notes | Field notes were collected after each interview |
| 21 | Duration | 19 min on average (range 8–53 min) |
| 22 | Data saturation | Discussed |
| 23 | Transcripts returned? | No |
| **Domain 3: Analysis and findings** | | |
| 25 | Number of data coders | 1 |
| 25 | Description of coding tree | No |
| 26 | Derivation of themes | Identified from the data |
| 27 | Software | NVivo |
| 28 | Participant checking | No |
| 29 | Quotation presented | Deidentified quotation were used to illustrate the themes |
| 30 | Consistent data findings | Clear link to data and findings |
| 31 | Clarity of major themes | Major themes were clearly presented in the results section |
| 32 | Clarity of minor themes | Description of diverse cases and discussion of minor themes included |
